# Prevalence of Bacterial Urinary Tract Infections and Associated Factors among Patients Attending Hospitals in Bushenyi District, Uganda

**DOI:** 10.1155/2019/4246780

**Published:** 2019-02-17

**Authors:** Martin Odoki, Adamu Almustapha Aliero, Julius Tibyangye, Josephat Nyabayo Maniga, Eddie Wampande, Charles Drago Kato, Ezera Agwu, Joel Bazira

**Affiliations:** ^1^Department of Microbiology and Immunology, Faculty of Biomedical Sciences, Kampala International University Western Campus, P.O. Box 71, Bushenyi, Kampala, Uganda; ^2^Department of Microbiology and Immunology, College of Health, Medicine and Life Sciences, St. Augustine International University, P.O. Box 88, Kampala, Uganda; ^3^Department of Biochemistry, Faculty of Biomedical Sciences, Kampala International University Western Campus, P.O. Box 71, Bushenyi, Kampala, Uganda; ^4^Department of Immunology and Molecular Biology, College of Health Sciences, Makerere University, P.O. Box 7072, Kampala, Uganda; ^5^School of Bio-security, Biotechnical and Laboratory Sciences, College of Veterinary Medicine, Animal Resources and Bio-security, Makerere University, P.O. Box 7062, Kampala, Uganda; ^6^Department of Microbiology, Faculty of Medicine, Mbarara University of Science and Technology, P.O. Box 1410, Mbarara, Uganda

## Abstract

Urinary tract infections (UTIs) are one of the major causes of morbidity and comorbidities in patients with underlying conditions, and it accounts for the majority of the reasons for hospital visit globally. Sound knowledge of factors associated with UTI may allow timely intervention that can easily bring the disease under control. This study was designed to determine the prevalence of UTI by isolating and characterizing the different bacterial etiological agents and to evaluate the factors associated with UTI. In this cross-sectional study, a total of 267, clean catch midstream urine (MSU) samples were collected aseptically and analyzed using standard microbiology methods. Data for the factors associated with UTI were obtained by use of questionnaires and standard laboratory tests for selected underlying conditions. The study revealed 86/267 (32.2%) UTI prevalence among patients attending hospitals in Bushenyi District, Uganda. *Escherichia coli* was the most prevalent bacterial uropathogen with 36/86 (41.9%) followed by *Staphylococcus aureus* 27/86 (31.4%), *Klebsiella pneumoniae* 10/86 (11.6%), *Klebsiella oxytoca* 6/86 (7.0%), *Proteus mirabilis* 3/86 (3.5%), *Enterococcus faecalis* 3/86 (3.5%), and *Proteus vulgaris* 1/86 (1.2%). This study has demonstrated that age ≤19 years, female gender, married individuals, genitourinary tract abnormalities, diabetes, hospitalization, indwelling catheter <6 days, and indwelling catheter >6 days had statistically significant relationships (*p* < 0.05) with UTI. Screening for UTI in hospitalized patients, female gender, married individuals, genitourinary tract abnormalities, indwelling catheter, and diabetics should be adopted.

## 1. Introduction

Urinary tract infections (UTIs) are the inflammatory disorders of the urinary tract caused by the abnormal growth of pathogens [1, 2]. Urinary tract infection is known to cause short-term morbidity in terms of fever, dysuria, and lower abdominal pain (LAP) and may result in permanent scarring of the kidney [3, 4]. Urinary tract infections can be community acquired or nosocomial. Community-acquired urinary tract infections (CA-UTIs) are defined as the infection of the urinary system that takes place in one's life in the community setting or in the hospital environment with less than 48 hours of admission. Community-acquired UTI is the second most commonly encountered microbial infection in the community setting [5]. Nosocomial urinary tract infections (N-UTIs) are the infection of the urinary tract that occurs after 48 hours of hospital admission, and the patient was not incubating at the time of admission or within 3 days after discharge [6].

Urinary tract infections may be asymptomatic, acute, chronic, and complicated or uncomplicated, and the clinical manifestations of UTIs depend on the portion of the urinary tract involved, the etiologic organisms, the severity of the infection, and the patient's ability to mount an immune response to it. Both asymptomatic and symptomatic UTIs pose a serious threat to public health care, hence reducing the quality of life and resulting into work absenteeism [7]. The symptoms of UTIs such as fever, burning sensations while urinating, LAP, itching, formation of blisters and ulcers in the genital area, genital and suprapubic pain, and pyuria generally depend on the age of the person infected and the location of the urinary tract infected [2].

Several factors such as gender, age, race, circumcision [8, 9], HIV [10–12], diabetes, urinary catheter, genitourinary tract abnormalities [13, 14], pregnancy, infants, elderly [15, 16], and hospitalization status [17] bear significant risk for recurrent UTIs. The commonest pathogenic organism isolated in UTI is *E. coli* followed by *K. pneumoniae*, *Staphylococcus*, *Proteus*, *Pseudomonas*, *Enterococcus*, and *Enterobacter* [18–21]. About 150 million people suffer from UTIs each year globally which results in greater than 6 billion dollars in direct health care [22]. The prevalence of UTIs in Algeria among all patients admitted in acute care units for more than 48 hours was reported to be 4.5% [23]. In Senegal, the prevalence was reported to be 0.7% among patients admitted in university hospital, Dakar Senegal, with a higher prevalence in women than males [23]. In Nigeria, in a study conducted among 12,458 urine samples, reported prevalence of community-acquired and nosocomial UTIs were 12.3% and 9.3%, respectively. The prevalence in females and the prevalence in males were 14.6% and 7.4%, respectively [24]. In Uganda, the prevalence of UTIs was found to be 29/218 (13.3%) and had a 20–60% drug resistance rate among antenatal mothers in Mulago hospital, Uganda [25]. Recently, UTIs were found to have a prevalence of 54/139 (38.8%), and age, female gender, and married individuals had statistical significant relations with the disease among adults attending the assessment centre, Mulago Hospital [26]. In Bushenyi District of Uganda, the prevalence of UTIs was 67/300 (22.33%) and *Escherichia coli* was the most prevalent bacterial uropathogen with 41/67 (61.19%) followed by *Staphylococcus aureus* 10/67 (14.93%), *Klebsiella pneumoniae* 4/67 (5.9%), *E. faecalis* 4/67 (5.6%), *M. morganii* 3/67 (4.8%), *Citrobacter species* 2/67 (2.99%), *Acinetobacter* 1 (1.49%), *Enterobacter* species 1 (1.49%), and *P. aeruginosa* 1/67 (1.49%) [27]. In a study of UTIs among diabetic individuals in Bushenyi District, Uganda, bacterial UTIs were 103/331 (31.1%) prevalent in diabetic patients and *Staphylococcus aureus* was the most prevalent bacterial uropathogen with 45/103 (43.7%) followed by *E. coli* 29/103 (28.2%), *Klebsiella* species 28/103 (27.2%), and *Enterococcus* species 1/103 (1.0%) [28]. To date, there is no detailed data from Bushenyi District, Uganda, that outlines the factors associated with urinary tract infections. This study was therefore designed to determine the etiology, factors associated with bacterial UTIs, and their strength among patients attending hospitals in Bushenyi District, Uganda.

## 2. Materials and Methods

### 2.1. Study Area

**Figure 1:**
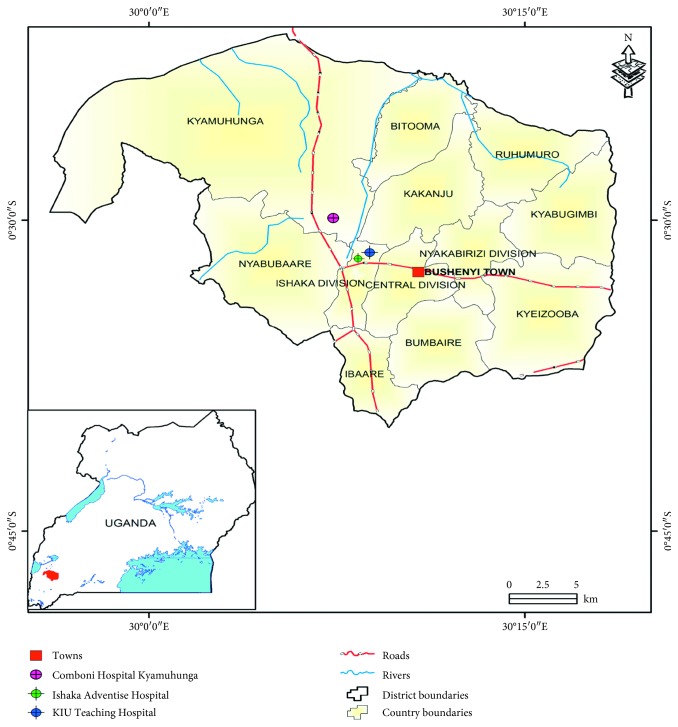
Map of Bushenyi District showing the study area (Uganda Bureau of Statistics, UBOS, 2015). Copyright © 1998–2018: Copyrights reserved to United Nations Office for the Coordination of Humanitarian Affairs, based on the OCHA/relief web.

This study was conducted in Bushenyi District. Geographically, Bushenyi District is located in the Western Region of Uganda. The district is composed of 9 subcounties, 3 divisions, 76 parishes, and 529 villages. According to the 2014 Uganda National Population Census, the population of Bushenyi district is 235,621 [29]. This population is served majorly by three hospitals: Kampala International University-Teaching Hospital (KIU-TH) which serves as a referral hospital in the district, Ishaka Adventist Hospital, and Comboni Hospital Kyamuhunga. These hospitals were chosen as the study sites because they are major health care providers for both outpatients and inpatients in the district (Figure 1).

### 2.2. Sample Size Determination

The sample size of 267 was arrived by use of the survey formula by Kish Leslie (1965); *n* = *z*^2^*p*(1 − *p*)/*d*^2^, where *z* = *Z* score for 95% confidence interval = 1.96, *p* = prevalence, and *d* = acceptable error (5%). We used the prevalence of UTIs among patients attending selected hospitals in Bushenyi district, Uganda, of 22.33% by Tibyangye et al. [27].

### 2.3. Study Design

This was a cross-sectional health-point survey conducted from June, 2017, to September, 2017. Both out and inpatients presenting or highly suspicious of having UTIs were recruited in the study. Only patients presenting or highly suspicious of having UTIs, living in Bushenyi district and attending treatment at Kampala International University-Teaching Hospital (KIU-TH), Ishaka Adventist Hospital and Comboni Hospital Kyamuhunga, were included in the study. Any patient who was terminally ill, who fails to give urine samples, with a history of antibiotic administration in the last two weeks and any female who was in their menstruation period were excluded from the study. Simple random sampling technique was applied to recruit patients who have satisfied the selection criteria from each hospital's outpatient and inpatient departments. Then, questionnaires with both open-ended questions such as age and closed ended questions with nominal categorical values such as gender were administered. Data including age, gender, tribe, residence, level of education, and history of medical conditions were collected by clinicians. Capillary blood and midstream urine (MSU) samples were collected after obtaining informed consent from the selected patients. Capillary blood was used for screening of selected factors associated with UTIs such as rapid HIV test, diabetic test, and only female patients' age ≥12 years underwent additional rapid pregnancy test, and the results were recorded accordingly.

### 2.4. Determination of HIV Status

In this test, one strip was used per individual. The patient's identification number was labeled on the test strip. A Pasteur or precision pipette was used to collect 50 μl of the specimen and only one drop of chase buffer was added to the specimen pad when using the blood specimen. The test results were read and recorded after 15 minutes (no longer than 60 minutes). Interpretation of the test result was done as follows: reactive: two lines of any intensity appear in both the control and patient test areas. Nonreactive: one line appears in the control area and no line in the patient test area. Invalid: no line appears in the control area. Invalid results were not reported. The tests were repeated with a new test strip if a line appeared in the patient area (determine HIV-1/2 Ag/Ab Combo Test, 2009). The determined positive HIV samples were confirmed using a Stat pack rapid test kit (Chembio Diagnostic, Inc., USA).

### 2.5. Determination of Diabetic Status

In this test, one strip was used per individual. The patient's identification number was labeled on the test strip. The test strip was dosed with 2.5 microliters of whole blood. The test result was read using an optium glucometer and recorded in 20 seconds. The normal standard reference ranges are random blood sugar (RBS) (3.3–7.4 mmol/l) or fasting blood sugar (FBS) (3.6–6.4 mmol/l) for adults. Children's fasting blood sugar (2.4–5.3 mmol/l) and new born's fasting blood sugar values are slightly lower (1.1–4.4 mmol/l). Diabetic mellitus diagnostic values when using capillary whole blood were follows: FBS is ≥ 6.7 mmol/l, and RBS is ≥ 11.1 mmol/l (Abbot Diabetes Care Ltd., UK).

### 2.6. Screening for Pregnancy

A human chronic gonadotropin (HCG) test was used to detect the presence of HCG hormones in females' age ≥12 years. The levels of HCG were detected using the early pregnancy test and ovulation predictors kits: FAQ (ACON Laboratories, Inc., USA). In this test, one strip was used per individual. The patient's identification number was labeled on the test strip. The test strip was dipped into a bottle of urine. Within 5 minutes, the result was read. Positive result: color bands appeared on both the test and control regions. Negative result: no color band appeared on the test region, and a color band appeared in the control region.

### 2.7. Urine Specimen Collection for Culture

A “clean catch” midstream urine sample was collected in sterile clean leak proof bottles from each patient. To avoid contamination of the specimen, all participants were required to first cleanse the urethral area with a castile soap towelette (Professional Disposables International, Inc., Canada). In addition, female participants were required to wide open the labia apart before sample collection. The MSU was then collected into a wide mouth clean sterile urine container. In patients with urinary catheters, urine specimens were collected from fresh catheters using a syringe and then transferred to a sterile specimen tube.

### 2.8. Isolation and Identification of Uropathogens

Isolation and identification of the bacterial uropathogens was done at Mbarara University of Science and Technology-Teaching Hospital (MUST-TH) microbiology laboratory. Each sample of the uncentrifuged, uniformly mixed MSU samples was inoculated on Cystine Lactose Electrolyte Deficient Agar (CLED) and incubated at 37°C aerobically for 24 hrs [30]. After incubation, the cultures were subcultured on MacConkey agar and Sheep Blood Agar (BA) media, observed, and recorded. Positive UTI was recorded after having presence of 100,000 colony-forming units (CFU) per milliliter in the culture of an appropriate collected MSU [31]. The isolates observed on the selective media were preserved in 40% glycerol at −80°C. For Gram-negative bacteria, standard identification procedures of colony morphology, gram staining, were followed by a subculture on the chromatic differential medium (Liofilchem, Italy) and use of the Analytical Profile Index (API 20E, BioMérieux, France) provided the presumptive identification of the pathogens [32]. The identity of the Gram-negative bacterial isolates was reported based on the discriminatory power of chromatic medium and API. The presumptive identity of the Gram-positive isolates was reported based on the phenotypic parameters like growth on mannitol salt agar (Oxoid, UK), chromatic agar, colony morphology, and Gram staining and then subsequent microscopical analysis and subjected to an appropriate biochemical test for proper identification. The identity of the Gram-positive isolates was done based on their cultural and biochemical characteristics as reported by Cheesbrough [30] and preserved in 40% glycerol at −80°C.

### 2.9. Data Analysis

Data analysis was done by descriptive statistics and regression using IBM SPSS version 20. Descriptive statistics was used to obtain UTI prevalence, uropathogens' frequency, and the mean age. The outcome of UTI was dichotomized as presence or absence of the disease and tested against suspected factors associated with UTI to assess for associations. Bivariate analysis was applied, and all the variables with a *p* value of 0.2 or less were entered into stepwise forward multiple logistic regression model. Interaction and confounding were assessed, and values of *p* ≤ 0.05 were regarded as statistically significant relationships.

### 2.10. Ethical Approval

The ethical approval of the study was sought from Mbarara University of Science and Technology (MUST, Institutional Research and Ethics Committee (IREC) on Human Research (no. 01/01-17), and the final approval was obtained from Uganda National Council for Science and Technology (UNCST) with UNCST Registration Number: HS 2232. All research protocols was performed in accordance with the ethical standards of committees on human experimentation laid down in the Helsinki declaration of 1964 revised in 2000 [33].

## 3. Results

### 3.1. Patients' Characteristics

Two hundred and sixty-seven (267) patients presenting or highly suspicious of having UTIs were recruited in the study upon obtaining informed consent and have met the selection criteria. The age of the patients was from 8 months to 95 years, and the mean of the study participants was 33.09 ± 23.731 years. The study participants were majorly females 176/267 (65.9%).

### 3.2. Prevalence of UTIs

Two hundred and sixty-seven (267) morning clean catch midstream urine samples were collected from patients attending three hospitals in Bushenyi district. Significant bacteriuria was observed in 86/267 (32.2%). The prevalence of bacterial UTI was highest in the age group 20–29 with 28/86 (32.6%) as compared to the lowest value of 1/86 (1.2%) in the adolescent age group of 10–19 years (Table 1). Urinary tract infection was highest in females with 66/176 (37.5%) as compared to 20/91 (22.0%) in men. *Escherichia coli* was the most prevalent bacterial uropathogen with 36/86 (41.9%) followed by *Staphylococcus aureus* 27/86 (31.4%), *Klebsiella pneumoniae* 10/86 (11.6%), *Klebsiella oxytoca* 6/86 (7.0%), *Proteus mirabilis* 3/86 (3.5%), *Enterococcus faecalis* 3/86 (3.5%), and *Proteus vulgaris* 1/86 (1.2%) (Table 2).

### 3.3. Factors Associated with UTIs

When the predictor variables for UTI were subjected to bivariate analysis, they had the following logistic regression values: hospitalization (OR = 4.002; 95% CI: 2.323–6.895; *p* < 0.05), age ≤19 years (OR = 0.359; 95% CI: 0.184–0.699; *p* < 0.05), female gender (OR = 2.130; 95% CI: 1.190–3.814; *p* < 0.05), married individuals (OR = 2.204; 95% CI: 1.203–4.037; *p* < 0.05), genitourinary abnormalities (OR = 2.387; 95% CI: 1.399–4.072; *p* < 0.05), catheter <6 days (OR = 2.730; 95% CI: 1.236–6.033; *p* < 0.05), catheter >6 days (OR = 8.604; 95% CI: 2.740–27.024; *p* < 0.05), and diabetes mellitus (OR = 2.738; 95% CI: 1.207–6.211; *p* < 0.05) were found to be statistically significant (*p* < 0.05) (Tables 3 and 4). When the bivariate significant predictor variables for UTI were subjected to multiple regression analysis, they had the following logistic regression values: hospitalization (OR = 3.633, 95% CI: 1.936–6.817; *p* < 0.05), female gender (OR = 2.521; 95% CI: 1.302–4.881; *p* < 0.05), catheter >6 days (OR = 3.761; 95% CI: 1.077–13.128; *p* < 0.05), genitourinary abnormalities (OR = 2.899; 95% CI: 1.597–5.262; *p* < 0.05), and diabetes mellitus (OR = 3.266; 95% CI: 1.292–8.256; *p* < 0.05) and were found to have statistically significant relationships (*p* < 0.05) with UTI (Table 5). However, residence, tribe, level of education, unmarried, circumcision, pregnancy, hypertension, HIV, abortion, sexual intercourse, and UTI symptoms were found to have no significant association with UTI.

## 4. Discussion

This study determined the prevalence, etiology, factors associated with bacterial UTI, and their strength among patients attending hospitals in Bushenyi District, Uganda. Our analysis demonstrated that the prevalence of bacterial UTI in Bushenyi District among patients attending hospitals was 86/267 (32.2%). Out of this bacterial UTI prevalence, symptomatic and asymptomatic patients contributed to 46/86 (53.5%) and 40/86 (46.5%), respectively. Almost half of the patients having significant bacteriuria were asymptomatic, and this situation is of utmost concern since asymptomatic bacteriuria is a strong predictor of ensuing symptomatic UTIs [34]. Previous study in Mulago by Mwaka et al. [35] found a much higher prevalence of significant bacteriuria of 29/40 (72.5%) in asymptomatic patients. The higher proportion in the study carried out at Mulago is not surprising, since the study included only adult females who are always at high risk of developing asymptomatic bacteriuria [26]. The prevalence of UTIs in this current study was found to be higher than the ones previously recorded in the following studies in Uganda: 67/300 (22.33%) by Tibyangye et al. [27] in Bushenyi District, 82/339 (24.2%) by Odongo et al. [36] in Gulu, 40/399 (10%) by Mwaka et al. [35] in Mulago, and more comparable to higher prevalence to 54/139 (38.8%) registered by Kabugo et al. [26] in Mulago hospital. The higher prevalence of UTIs in our study could have been probably due to the inclusion of a number of risk groups like diabetes, elderly, pregnant women, HIV, infants, and a high number of inpatients who are usually prone to UTIs.

Our study demonstrated *E. coli* as the most prevalent bacterial uropathogen with 36/86 (41.9%). This finding is comparable with other studies elsewhere in Africa indicating 40–46% of isolation of *E. coli* [37–40]. The high prevalence of 27/66 (40.9%) of *E. coli* in the female gender could be due to the close proximity of the anus to the vagina. This high possibility of UTIs in females is due to the inherent virulence of *E. coli* for urinary tract colonization such as its abilities to adhere to the urinary tract and also association with other microorganisms moving from the perineum areas contaminated with fecal microbes to the moist warmth environment of the female genitalia [25, 41]. *Staphylococcus aureus* was the second most isolated bacterial uropathogen with 27/86 (31.4%) of frequency. The high frequency of *S. aureus* in UTI is not unique to this study. Earlier studies in Bushenyi (Uganda) 2015, Mulago (Uganda) 2011, and Awka (Nigeria) 2016 reported high rates of *S. aureus* of 45/103 (43.7%), 9/40 (22.5%), and 60/215 (28%), respectively [28, 35, 42]. Previous studies have linked the increasing *Staphylococcal* UTIs to increased use of instrumentation such as bladder catheters [43, 44]. However, the high prevalence of *Staphylococcus* in this study varied from other previous studies [1, 45, 46]. However, the isolation of *Klebsiella pneumoniae* 10/86 (11.6%), *Klebsiella oxytoca* 6/86 (7.0%), and *Proteus mirabilis* 3/86 (3.5%) is in agreement with other studies by Baguma et al. [47] in Southwestern Uganda and Lo et al. in São Paulo Brazil [48]. The other isolates in this study included *Enterococcus faecalis* 3/86 (3.5%) and *Proteus vulgaris* 1/86 (1.2%) which is comparable with other studies done by Khanal et al. [49] in Nepal and Lo et al. [48] in São Paulo Brazil.

This study demonstrated that age ≤19 years, female gender, married individuals, diabetes, genitourinary tract abnormalities, hospitalization, catheter, and increase in duration of catheter were found to bear statistically significant relationship with UTIs. Age and female gender were found to have statistically significant relationship with UTIs in similar study carried out by Kabugo et al. in 2016 [26] at Mulago hospital in Uganda. The statistically significant association between UTIs and diabetes could be due to altered immunity in diabetic patients which includes depressed polymorphonuclear leukocyte functions, altered leukocyte adherence, chemotaxsis, phagocytosis, impaired bactericidal activity of the antioxidant system [50, 51], and neuropathic complications, such as impaired bladder emptying. In addition, a higher glucose concentration in the urine may create a culture medium for pathogenic microorganisms in diabetic patients that may result into UTIs. Generally, similar reports from elsewhere also indicated that age, female gender [26, 52], genitourinary tract abnormalities [13, 14], diabetes [28, 52, 53], married individuals [54], hospitalization [17], catheter, and duration of catheter [52] bear statistically significant relationship with UTIs. The high prevalence of bacteriuria among inpatients, 49/86 (57.0%), as compared to the outpatients, 37/86 (43.0%), was due to increased risk of infection due to indwelling catheter that contributed to 59.2% of the inpatients' UTIs. This finding is in agreement with the previous study done by Adukauskiene et al. [17].

## 5. Conclusion

In this study, the prevalence of UTIs from patients attending hospitals in Bushenyi District, Uganda, was found to be 86/267 (32.2%). *Escherichia coli* and *S. aureus* are the major causes of both CA-UTIs and N-UTIs among patients attending hospitals in Bushenyi District, Uganda. This study has demonstrated that hospitalization, married individuals, duration of catheter, diabetes mellitus, genitourinary tract abnormalities, and female gender are the most important factors associated with UTIs. Appropriate measures may help to reduce UTIs due to these associated factors. We recommend routine UTIs screening of patients of the following category: hospitalized, genitourinary tract abnormalities, indwelling catheter, diabetic, female gender, and married individuals. If these routine checks are put in place, prevention of UTI can be realized at lower cost.

## Figures and Tables

**Table 1:** Age-specific prevalence of bacterial uropathogens.

Age range	Positive UTI (%)	Negative UTI (%)	Total (%)
<1	7 (8.1)	13 (7.2)	20 (7.5)
1–9	5 (5.8)	28 (15.5)	33 (12.4)
10–19	1 (1.2)	19 (10.5)	20 (7.5)
20–29	28 (32.6)	36 (19.9)	64 (24.0)
30–39	10 (11.6)	22 (12.2)	32 (12.0)
40–49	10 (11.6)	20 (11.0)	30 (11.2)
50–59	8 (9.3)	19 (10.5)	27 (10.1)
≥60	17 (19.8)	24 (13.3)	41 (15.4)
Total	86 (100)	181 (100)	267 (100)

**Table 2:** Prevalence of bacterial uropathogen isolates from MSU.

Uropathogens	Male, *n* (%)	Female, *n* (%)	Total, *n* (%)
*E. coli*	9 (45.0)	27 (40.9)	36 (41.9)
*S. aureus*	4 (20.0)	23 (34.8)	27 (31.4)
*K. pneumoniae*	3 (15.0)	7 (10.6)	10 (11.6)
*K. oxytoca*	1 (5.0)	5 (7.6)	6 (7.0)
*P. mirabilis*	1 (5.0)	2 (3.0)	3 (3.5)
*E. faecalis*	2 (10.0)	1 (1.5)	3 (3.5)
*P. vulgaris*	0 (0.0)	1 (1.5)	1 (1.2)
Total	20 (100)	66 (100)	86 (100)

**Table 3:** Bivariate analysis between sociodemographic variables and UTI.

Variables	Categories	Unadjusted odds ratio	95% CI	*p* value
Department	Inpatients	4.002	2.323–6.895	**0.000**
	Outpatients	1		
Age	≤19 years	0.359	0.184–0.699	**0.003**
	≥20 years	1		
Gender	Female	2.130	1.190–3.814	**0.011**
	Male	1		
Residence	Rural	0.736	0.409–1.324	0.306
	Suburban	1.115	0.499–2.490	0.790
	Urban	1		
Tribes	Bairu	0.454	0.154–1.341	0.153
	Bakiga	4.043	0.455–35.948	0.210
	Bahima	1.692	0.165–17.393	0.658
	Baganda	0.124	0.011–1.367	0.088
	Others	1		
Marital status	Married	2.204	1.203–4.037	**0.011**
	Single	0.393	0.110–1.408	0.151
	Others	1		
Level of education	No education	1.165	0.632–2.148	0.624
	Primary	1.242	0.610–2.529	0.550
	Secondary	0.828	0.375–1.829	0.640
	Tertiary	1		
Circumcision	Yes	0.382	0.080–1.824	0.228
	No	1		
Sexual intercourse	Yes	0.825	0.432–1.574	0.559
	No	1		

CI = confidence interval; *p* = probability; *p* ≤ 0.05 value is statistically significant under logistic regression.

**Table 4:** Bivariate analysis between health condition and UTI.

Variables	Categories	Unadjusted odds ratio	95% CI	*p* value
Pregnancy	Yes	1.076	0.544–2.128	0.834
	No	1		
Hypertension	Yes	1.658	0.788–3.489	0.183
	No	1		
Genitourinary abnormalities	Yes	2.387	1.399–4.072	**0.001**
	No	1		
Catheters <6 days	Yes	2.730	1.236–6.033	**0.013**
	No	1		
Catheters >6 days	Yes	8.604	2.740–27.024	**0.000**
	No	1		
Diabetes mellitus	Yes	2.738	1.207–6.211	**0.016**
	No	1		
HIV	Yes	1.144	0.537–2.438	0.728
	No	1		
Abortion	Yes	1.574	0.558–4.443	0.391
	No	1		
UTI symptoms	Yes	0.692	0.412–1.164	0.165
	No	1		

CI = confidence interval; *p* = probability; *p* ≤ 0.05 value is statistically significant under logistic regression.

**Table 5:** Factors associated with UTI using stepwise forward multiple logistic regression analysis.

Factors associated with UTI	Adjusted odds ratio	95% CI	*p* value
Inpatients	3.633	1.936–6.817	**0.000**
Female	2.521	1.302–4.881	**0.006**
Catheters >6 days	3.761	1.077–13.128	**0.038**
Genitourinary abnormalities	2.899	1.597–5.262	**0.000**
Diabetes mellitus	3.266	1.292–8.256	**0.012**

CI = confidence interval; *p* = probability; *p* ≤ 0.05 value is statistically significant under logistic regression.

## Data Availability

The data in tables used to support the findings of this study are included within the article.

## References

[1] Prakash D., Saxena R. S. (2013). Distribution and antimicrobial susceptibility pattern of bacterial pathogens causing urinary tract infection in Urban Community of Meerut City, India. *ISRN Microbiology*.

[2] Amali O., Indinyero M. D., Umeh E. U., Awodi N. O. (2009). Urinary tract infections among female students of the university of agriculture, Makurdi, Benue State, Nigeria. *Internet Journal of Microbiology*.

[3] Hoberman A., Charron M., Hickey R. W., Baskin M., Kearney D. H., Wald E. R. (2003). Imaging studies after a first febrile urinary tract infection in young children. *New England Journal of Medicine*.

[4] Camacho V., Estorch M., Fraga G. (2004). DMSA study performed during febrile urinary tract infection: a predictor of patient outcome?. *European Journal of Nuclear Medicine and Molecular Imaging*.

[5] Sabrina J. (2010). Antimicrobial resistance among producers and non-producers of extended spectrum beta-lactamases in urinary isolates at a tertiary Hospital in Tanzania. *BMC Research Notes*.

[6] Lacovelli V., Gaziev G., Topazio L., Bove P., Vespasiani G., Finazzi A. E. (2014). Nosocomial urinary tract infections: a review. *Urologia*.

[7] Olowe O., Ojo-Johnson B., Makanjuola O., Olowe R., Mabayoje V. (2015). Detection of bacteriuria among human immunodeficiency virus seropositive individuals in Osogbo, south-western Nigeria. *European Journal of Microbiology and Immunology*.

[8] Conway P. H., Cnaan A., Zaoutis T., Henry B. V., Grundmeier R. W., Keren R. (2007). Recurrent urinary tract infections in children: risk factors and association with prophylactic antimicrobials. *JAMA*.

[9] Dias C. S., Silva J. M. P., Diniz J. S. S. (2010). Risk factors for recurrent urinary tract infections in a cohort of patients with primary vesicoureteral reflux. *Pediatric Infectious Disease Journal*.

[10] Banu A., Jyothi R. (2013). Asymptomatic bacteriuria in HIV positive individuals in a tertiary care hospital. *Journal of HIV and Human Reproduction*.

[11] Iduoriyekemwen N. J. S. W., Sadoh A. E. (2012). Asymptomatic bacteriuria in HIV positive Nigerian children. *Journal of Medicine and Biomedical Research*.

[12] Ibadin O. M., Onunu A., Ukoh G. (2006). Urinary tract infection in adolescent/young adult Nigerians with acquired human immuno deficiency disease in Benin city. *JMBR: Journal of Biomedical Sciences*.

[13] Mladenovic J., Veljovic M., Udovicic I. (2015). Catheter-associated urinary tract infection in a surgical intensive care unit. *Vojnosanitetski Pregled*.

[14] Yuyun M. F., Angwafo F. F., Koulla-Shiro S., Zoung-Kanyi J. (2004). Urinary tract infections and genitourinary abnormalities in Cameroonian men. *Tropical Medicine and International Health*.

[15] Nicolle L. E. (2008). Uncomplicated urinary tract infection in adults including uncomplicated pyelonephritis. *Urologic Clinics of North America*.

[16] Nelson J. M., Good E. (2015). Urinary tract infections and asymptomatic bacteriuria in older adults. *Nurse Practitioner*.

[17] Adukauskiene D., Cicinskaite I., Vitkauskiene A., Macas A., Tamosiunas R., Kinderyte A. (2006). Hospital acquired urinary tract infections. *Medicinia (Kaunas)*.

[18] Manges A. R., Natarajan P., Solberg O. D., Dietrich P. S., Riley L. W. (2006). The changing prevalence of drug-resistant Enterobacteriaceae groups in a community: evidence for community outbreaks of urinary tract infections. *Epidemiology and Infections*.

[19] Akram M., Shahid M., khan A. (2007). Etiology and antibiotic resistance pattern of community acquired urinary tract infection in JNMC Hospital India. *Annals of Clinical Microbiology and Antimicrobia*.

[20] E E. A., O K. I. (2008). Incidence and antibiotic susceptibility pattern of *Staphylococcus aureus* amongst patients with urinary tract infection (UTI) in UBTH Benin City, Nigeria. *African Journal of Biotechnology*.

[21] Mirsoleymani S. R., Salimi M., Shareghi B. M., Ranjbar M., Mehtarpoor M. (2014). Bacterial pathogens and antimicrobial resistance patterns in pediatric urinary tract infections: a four-year surveillance study (2009–2012). *International Journal of Pediatrics*.

[22] Stamm W. E., Norrby S. R. (2001). Urinary tract infections: disease panorama and challenges. *Journal of Infectious Diseases*.

[23] Nejad S. B., Allegranzi B., Syed S., Ellis B., Pittet D. (2011). Health-care-associated infection in Africa: a systematic review. *Bulletin of the World Health Organization*.

[24] Jombo G. T., Egah D. Z., Banwat E. B., Ayeni J. A. (2006). Nosocomial and community acquired urinary tract infections at a teaching hospital in north central Nigeria: findings from a study of 12,458 urine samples. *Nigerian Journal of Medicine*.

[25] Andabati G., Byamugisha J. (2010). Microbial aetiology and sensitivity of asymptomatic Bacteriuria among ante-natal mothers in Mulago hospital, Uganda. *African Health Sciences*.

[26] Kabugo D., Kizito S., Ashok D. D. (2016). Factors associated with community-acquired urinary tract infections among adults attending assessment centre, Mulago Hospital Uganda. *African Health Sciences*.

[27] Tibyangye J., Okech M., Nyabayo J., Nakavuma J. (2015). *In vitro* antibacterial activity of *Ocimum suave* essential oils against uropathogens isolated from patients in selected hospitals in Bushenyi district, Uganda. *British Microbiology Research Journal*.

[28] Odoki M., Bazira J., Moazam M. L., Agwu E. (2015). Health-point survey of bacteria urinary tract infections among suspected diabetic patients attending clinics in Bushenyi district of Uganda. *Special Bacterial Pathogens Journal (SBPJ)*.

[29] UBOS (2014). *The Population of the Regions of the Republic of Uganda and All Cities and Towns of More*.

[30] Cheesbrough M. (2006). *District Laboratory Practice in Tropical Countries*.

[31] Harding G. K. M., Zhanel G. G., Nicolle L. E., Cheang M., The Manitoba Diabetes Urinary Tract Infection Study Group (2002). Antimicrobial treatment in diabetic women with asymptomatic bacteriuria. *New England Journal of Medicine*.

[32] Holmes B., Willcox W. R., Lapage S. P. (1978). Identification of enterobacteriaceae by the API 20E system. *Journal of Clinical Pathology*.

[33] World Medical Association Declaration of Helsinki (2000). *Ethical Principles for Medical Research Involving Human Subjects*.

[34] Hooton T. M., Scholes D., Stapleton A. E. (2000). A prospective study of asymptomatic bacteriuria in sexually active young women. *New England Journal of Medicine*.

[35] Mwaka A. D., Mayanja-Kizza H., Kigonya E., Kaddu-Mulindwa D. (2011). Bacteriuria among adult non-pregnant women attending Mulago hospital assessment centre in Uganda. *African Health Sciences*.

[36] Odongo C. O., Anywar D. A., Luryamamoi K., Odongo P. (2013). Antibiograms from community-acquired uropathogens in Gulu, northern Uganda—a cross-sectional study. *BMC Infectious Diseases*.

[37] Kayima J. K., Otieno L. S., Twahir A. (1996). Asymptomatic bacteriuria among diabetics attending Kenyatta National Hospital. *East Afr. Med. J.*.

[38] Moges A. F., Genetu A., Mengistu G. (2002). Antibiotic sensitivities of common bacterial pathogens in urinary tract infections in Gondar Hospital, Ethiopia. *East African Medical Journal*.

[39] Wanyama J. (2003). Prevalence, bacteriology and microbial sensitivity patterns among pregnant women with clinically diagnosed urinary tract infections in Mulago Hospital Labour Ward.

[40] Mayanja R., Kiggundu C., Kaddu-Mulindwa D. (2005). The prevalence of asymptomatic bacteriruria and associated factors among women attending antenatal clinics in lower Mulago Hospital.

[41] McLaughlin S. P., Carson C. C. (2004). Urinary tract infections in women. *Medical Clinics of North America*.

[42] Ekwealor P. A., Ugwu M. C., Ezeobi I. (2016). Antimicrobial evaluation of bacterial isolates from urine specimen of patients with complaints of urinary tract infections in Awka, Nigeria. *International Journal of Microbiology*.

[43] Moore K. N., Day R. A., Albers M. (2002). Pathogenesis of urinary tract infections: a review. *Journal of Clinical Nursing*.

[44] Iregbu K., Nwajiobi-Princewill P. (2013). Urinary tract infections in a tertiary hospital in Abuja, Nigeria. *African Journal of Clinical and Experimental Microbiology*.

[45] Ochada N., Nasiru I., Thairu Y., Okanlowan M., Abdulakeem Y. (2014). Antimicrobial susceptibility pattern of urinary pathogens isolated from two tertiary hospitals in Southwestern Nigeria. *African Journal of Clinical and Experimental Microbiology*.

[46] Bano K., Khan J., Begum R. H. (2012). Patterns of antibiotic sensitivity of bacterial pathogens among urinary tract infections (UTI) patients in a Pakistani population. *African Journal of Microbiological Research*.

[47] Baguma A., Atek K., Bazira J. (2017). Prevalence of extended-spectrum beta-lactamases-producing microorganisms in patients admitted at KRRH, southwestern Uganda. *International Journal of Microbiology*.

[48] Lo D. S., Shieh H. H., Ragazzi S. L. B., Koch V. H. K., Martinez M. B., Gilio A. E. (2013). Community-acquired urinary tract infection: age and gender-dependent etiology. *Jornal Brasileiro de Nefrologia*.

[49] Khanal L. K., Shrestha R., Barakoti A., Timilsina S., Amatya R. (2016). Urinary tract infection among males and females- a comparative study. *Nepal Medical College Journal*.

[50] Stapleton A. (2002). Urinary tract infections in patients with diabetes. *American Journal of Medicine*.

[51] Hopps E., Camera A., Caimi G. (2008). Polymorphonuclear leukocytes and diabetes mellitus. *Minerva Medica*.

[52] Ally S. A., Tawfeek R. A., Mohamed I. S. (2016). Bacterial catheter-associated urinary tract infection in the intensive care unit of assiut university hospital. *Al-Azhar Assiut Medical Journal*.

[53] Simkhada R. (2013). Urinary tract infection and antibiotic sensitivity among diabetics. *Nepal Medical College Journal*.

[54] Angus N. O., Vivian B. A., Chijioke E. E. (2017). Bacteriology and antibiogram of urinary tract infection among female patients in a tertiary health facility in south eastern Nigeria. *Open Microbiology Journal*.

